# Effects of home quarantine during COVID-19 lockdown on physical activity and dietary habits of adults in Saudi Arabia

**DOI:** 10.1038/s41598-021-85330-2

**Published:** 2021-03-15

**Authors:** Hanan Alfawaz, Osama E. Amer, Abdulaziz A. Aljumah, Dara A. Aldisi, Mushira A. Enani, Naji J. Aljohani, Naif H. Alotaibi, Naemah Alshingetti, Suliman Y. Alomar, Malak Nawaz Khan Khattak, Shaun Sabico, Nasser M. Al-Daghri

**Affiliations:** 1 Chair for Biomarkers of Chronic Diseases, Biochemistry Department, College of Science, King Saud University, PO Box 2455, Riyadh, 11451 Saudi Arabia; 2 College of Food Science and Agriculture, Department of Food Science and Nutrition, King Saud University, Riyadh, 11495 Saudi Arabia; 3 College of Medicine Medical Student, Almaarefa University, Riyadh, 11597 Saudi Arabia; 4 Department of Community Health Sciences, College of Applied Medical Sciences, King Saud University, Riyadh, 11451 Saudi Arabia; 5 Medical Specialties Department, King Fahad Medical City, Riyadh, 59046 Saudi Arabia; 6 Obesity, Endocrine and Metabolism Center, Department of Medicine, King Fahad Medical City, Riyadh, Saudi Arabia; 7 Department of Medicine, College of Medicine, King Saud University, Riyadh, Saudi Arabia; 8 Obstetrics and Gynaecology Department, King Salman Bin Abdulaziz Hospital, Riyadh, 11564 Saudi Arabia; 9 Doping Research Chair, Department of Zoology, College of Science, King Saud University, Riyadh, 11495 Saudi Arabia

**Keywords:** Health care, Public health, Epidemiology

## Abstract

Public health endorsements during the present COVID-19 pandemic has led the governments of largely affected countries to imply policies that restrict social mobility to slow COVID-19 spread. The study aimed to explore the effects of COVID-19 home quarantine on lifestyle and health behavior of Saudi residents. An online survey in Saudi Arabia was launched from May 11 to June 6, 2020. The survey was designed by multidisciplinary scientists and academics uploaded and shared through the Google platform in Arabic and English languages. Questions presented related to responses “before” and “during” COVID-19 home quarantine. A total of 1965 respondents participated and were included in the analysis [921 (47.0%) males and 1044 (53.0%) females]. Non-Saudis were more likely to increase their physical activity during quarantine [odds ratio (95% confidence interval 1.41 (1.11–1.79); *p* < 0.005]. Prevalence of participants walking daily for more than 4 times per week significantly decreased during pandemic (before vs during, 30.5% vs 29.1%) which was in parallel to the significant increase in the prevalence of participants who did not perform daily walking during the quarantine (21% vs 22.9%; *p* < 0.001). The prevalence of participants who often consume snacks between meals increased during quarantine (27.4% vs 29.4%, *p* < 0.001), while the prevalence of participants who never consumed fresh fruits and vegetables significantly increased during home quarantine (2.4% vs 3.7%; *p* = 0.019). The lockdown imposed in Saudi Arabia modestly but significantly impacted physical activity and dietary behaviors of several citizens and residents in an unhealthy way. Interventions to alleviate these acute adverse lifestyle behaviors during pandemic should be formulated.

## Introduction

The present pandemic of COVID-19 has caused governments of largely affected countries to force harsh lockdown precautionary rules on their citizens. These included working from home, closing shops, schools, restaurants and any non-essential service or business to slow down the spread of the outbreak and prevent health care system collapse. These quarantine rules resulted in unhealthy behaviors which adversely affected the general population health^1,2^. Furthermore, restrictions in physical activity was due to closed sport centers/gyms and limited social mobility^3^. These limitations may aggravate sedentary lifestyle, an unhealthy habit highly prevalent in developed nations, including Saudi Arabia^4^. In fact, before the pandemic, being sedentary was already deemed a major public health problem, with more than a quarter of all adults not meeting the required physical activity levels for maintaining good health^5^. During the pandemic however, a recently released data for physical activity by Fitbit, Inc., indicated that the average step counts of more than 30 million users and showed a significant decline in step count which varied across nations, ranging from 7 to 38% decrease during the week ending March 22, 2020, as compared with the same period last year^6^. This observation suggested that isolation may provoke an extensive reduction in physical activity levels as observed in other confined situations like incarceration and space travel^7,8^. Given that a chronic sedentary lifestyle is detrimental for health^9^, light weight activities come into play, as decreasing sitting times is similarly important as increasing exercising periods^10^.

Other consequences of prolonged quarantine include altered nutritional intake, e.g., overeating^11,12^. During quarantine, our diet is altered to include foods with a long shelf-life, again, due to limited access for outdoor food purchases^11^. Additionally, many people typically respond to chronic stressful conditions by seeking out foods that are dense in energy^13–16^. Torres and colleagues observed that people tend to alleviate the lockdown-related stress by having a higher than normal consumption of less nutritious but comforting foods (e.g., hamburgers, snacks, chocolate, and carbonated beverages) in an attempt to feel better (stress-related eating)^17^. Furthermore, isolation during quarantine harbors lack of emotional support from relatives and friends which is predictive of stress-driven eating and drinking behaviors^18,19^.

Most of the COVID-19 studies in Saudi Arabia have focused on pre-existing conditions with final outcomes^20,21^, as well as dietary changes and mental health^22,23^. Given that lockdowns primarily promote a more sedentary and passive lifestyle, the present study aimed to determine the extent of altered lifestyle behavior changes during this period through a structured online survey distributed to residents of Saudi Arabia.

## Methods

### Study design and participants

This cross-sectional survey was conducted from May 11 to June 6, 2020, corresponding to 2 weeks during and after the holy month of Ramadan (April 23-May 23, 2020), using an online electronic survey (supplementary file 1). Within this timeframe, confirmed Covid-19 cases in Saudi Arabia increased from 41,014 to 98,869 (John Hopkins Corona Center; https://coronavirus.jhu.edu/)^24^. Table 1 shows the timeline of events with respect to restrictions imposed in Saudi Arabia and the questionnaire respondents for each event. Majority of the respondents answered the questionnaire during Ramadan (75.1%). All adult Saudi citizens and residents (non-Saudis) 18 years old and above with access to internet were deemed eligible to voluntarily participate in the survey. All participants were asked for their email addresses for verification purposes only and to ensure that each participant completed the survey only once. The study design and protocol were approved by the Ethics Committee for Scientific Research and Post Graduate Studies at the College of Science, King Saud University, Saudi Arabia (reference# KSU-HE-20-246). Informed consent was obtained from all respondents. All methods were carried out in accordance with relevant guidelines and regulations.

**Table 1:** Timeline of Events.

Event	Dates	Curfew hours	Exceptions	N (%)
Suspension of non-essential work	Mar 15		None	
Nationwide curfew	Mar 23–Apr 5	6AM–7PM	Makkah/Madinah	
Enhanced curfew	Apr 6–25	6AM–3PM	None	
Ramadan^a^	Apr 26–May 22	9AM–5PM	Makkah	1476 (75.1)
Eid Al Fitr^a^	May 23–27	24 h	None	422 (21.5)
Phase 1 partial easing^a^	May 28–30	6AM–3PM	Makkah	56 (2.8)
Phase 2 partial easing^a^	May 31–Jun 20	6AM–6PM	Makkah	11 (0.6)

^a^Denotes events that unfolded during the study period (May 11–June 6, 2020).

### Questionnaire

The questionnaire included a cover letter in Arabic and English. It consisted of demographic and social information, general awareness about the pandemic and statements in Likert scale format to determine changes in behavioral lifestyle, dietary habits, physical activity, and mental wellness among others. Experts in the related field reviewed the questionnaire and several revisions were made to strengthen the reliability and validity of the questionnaire as well as enhance scientific value of the data to be collected. A pilot study (n = 75 participants) was performed to confirm reliability and validity of the questionnaire and obtained Cronbach’s α, which was noted to be excellent (overall 84%, demographics 88%, physical activity 81% and dietary habits 91%). After completion, the questionnaire was cascaded to different social media outlets throughout Saudi Arabia.

### Sample size calculation

Sample size calculation was done using Raosoft online to specify the number of respondents needed with an error margin to meet the desired confidence level. To obtain a confidence level of 95% and a 2.2% margin of error, a minimum sample size of 1946 would enable us to achieve the study objectives.

### Data analysis

Analysis was done using SPSS version 16.5 (Chicago, IL, USA). Continuous variables were presented as mean ± standard deviation while categorical variables were presented as frequencies (N) and percentages (%). Chi-Square test was used to determine differences between categorical variables of interest. Independent T-test was used to determine differences between normal continuous variables and Mann–Whitney U-test for non-normal continuous variables. Cochran’s Q test was used to assess the main effects of groups over time before and during the pandemic. Post-hoc comparisons between scales was done using McNemar’s test. Multinomial logistic regression analysis was performed for physical activities change for independent predictors. Assumptions on independence within observations, uncorrelated errors and consistency in coding were confirmed prior to reliability analysis (Cronbach α > 0.7). *p* value was considered significant at *p* < 0.05.

## Results

### Sample description

This cross-sectional study included a total of 1,965 respondents [males, n = 921 (46.9%) and females, n = 1044 (53.1%) participants], all of whom completed the entire questionnaire. Table 2 shows the demographic characteristics of the participants.

**Table 2:** Demographic characteristics of participants.

Parameters	All	Males	Females	*p* value
N (%)	1965	921 (46.9)	1044 (53.1)	
Age* (year)(min–max)	35.2 ± 13.1 (15–75)	35.2 ± 13.1 (16–72)	35.1 ± 12.9 (15–70)	0.80
15–25 year	609 (31.0)	292 (31.7)	317 (30.4)	0.49
26–35 year	516 (26.3)	231 (25.1)	285 (27.3)	
36–45 year	411 (20.9)	187 (20.3)	224 (21.5)	
> 45 year	429 (21.8)	211 (22.9)	218 (20.9)	
**Marital status (%)**				
Single	860 (43.8)	355 (38.5)	509 (48.8)	< 0.001
Married	1040 (52.9)	566 (61.6)	474 (45.4)	
Divorce	49 (2.5)	0 (0.0)	49 (4.7)	
Widow/widower	12 (0.6)	0 (0.0)	12 (1.1)	
**Nationality (%)**				
Saudi	1632 (83.1)	777 (84.4)	855 (81.9)	0.15
Non-Saudi	333 (16.9)	144 (15.6)	189 (18.1)	
**Education (%)**				
High school	165 (8.4)	75 (8.1)	90 (8.6)	0.36
Bachelor	1158 (58.9)	556 (60.4)	602 (57.7)	
Master	368 (18.7)	158 (17.2)	210 (20.1)	
PHD	274 (13.9)	132 (14.3)	142 (13.6)	
**Family monthly income (SAR) (%)**				
< 5000	687 (35.0)	338 (36.7)	349 (33.4)	0.42
5000–7000	200 (10.2)	93 (10.1)	107 (10.2)	
8000–16,000	580 (29.5)	258 (28.0)	322 (30.8)	
> 16,000	498 (25.3)	232 (25.2)	266 (25.5)	
**Employment status (%)**				
Employed	1100 (56.0)	495 (53.7)	605 (58.0)	0.30
Unemployed	217 (11.0)	113 (12.3)	104 (10.0)	
Student	597 (30.4)	286 (31.1)	311 (29.8)	
Own business	2 (0.1)	1 (0.1)	1 (0.1)	
Farmer	49 (2.5)	26 (2.8)	23 (2.2)	
**Family members (%)**				
02-Apr	545 (27.7)	261 (28.3)	284 (27.2)	0.82
04-Jun	700 (35.6)	319 (34.6)	381 (36.5)	
> 6	587 (29.9)	276 (30.0)	311 (29.8)	
Single	133 (6.8)	65 (7.1)	68 (6.5)	

Data presented as n (%); *denotes data presented as mean ± standard deviation.

*p* value significant at < 0.05.

Table 3 shows physical activity information from all responses before and during home quarantine. Over-all significant differences were observed in between group comparisons over time for physical activity in terms of walking, home physical activities with weights and swimming (*p* values < 0.001). In the daily walking physical activities, the percentage of participants walking daily for more than 4 times per week significantly decreased during pandemic (before vs during, 30.5% vs 29.1%; *p* < 0.05) which was in parallel to the significant increase in the percentage of participants who did not perform daily walking during the quarantine (21% vs 23.6%; *p* < 0.001). Similarly, the percentage of participants who never performed home physical activities with weights before the quarantine significantly increased during the quarantine (42.8% vs 44.6%; *p* < 0.001). In contrast, there was a significant percentage of participants who increased their frequency by 3–4 times per week in swimming during the quarantine (3% vs 4.6%; *p* < 0.001).

**Table 3:** Responses to the physical activity questionnaire recorded before and during home quarantine.

Physical activity	Days per week [n (%)]								*p* value
	Before				During				
	Never	1–2	3–4	> 4	Never	1–2	3–4	> 4	
Daily walking	413 (21.0)	463 (23.6)	489 (24.9)	600 (30.5)	464 (23.6)**	450 (22.9)	479 (24.4)	572 (29.1)*	< 0.001
Home physical activities with weights	842 (42.8)	374 (19.0)	354 (18.0)	395 (20.1)	877 (44.6)**	369 (18.8)	336 (17.1)	383 (19.5)	< 0.001
Swimming	1737 (88.4)	128 (6.5)	58 (3.0)	42 (2.1)	1693 (86.2)**	132 (6.7)	90 (4.6)**	50 (2.5)	< 0.001

Data presented n (%).

* and ** represented *p* value significant at < 0.05 and 0.01 level using Cochran’s Q test and McNemar’s test.

Table 4 demonstrates the unadjusted odds ratios (ORs) with confidence intervals (95% CI) of factors affecting physical activity. In general, physical activity was highest among: Non-Saudis [OR 1.41 (1.11–1.79); *p* = 0.005], high income [1.45 (1.05–1.99); < 0.05] and middle age [1.57 (1.22–2.01); *p* = 0.001]. Physical activity was lowest among respondents with master’s degree [0.58 (0.40–0.84); *p* = 0.004]. Non-Saudis were more likely to increase their physical activity during quarantine.

**Table 4:** Factors affecting physical activity.

Categories	All	*p* value
	Crude OR (95%CI)	
**Marital status**		
Single	1	0.22
Married	1.12 (0.93–1.34)	0.80
Divorce	0.93 (0.52–1.65)	0.44
Widow	0.64 (0.20–2.02)	
**Nationality**		
Saudi	1	**0.005**
Non-Saudi	1.41 (1.11–1.79)	
**Education**		
High school	1	0.21
Bachelor	0.81 (0.58–1.13)	**0.004**
Master	0.58 (0.40–0.84)	0.48
PHD	1.15 (0.78–1.71)	
**Family monthly income**		
< 5000	1	**0.02**
5000–7000	1.45 (1.05–1.99)	0.13
8000–16,000	1.19 (0.95–1.48)	**0.02**
> 16,000	1.31 (1.04–1.65)	
**Employment status**		
Employed	1	0.91
Unemployed	0.98 (0.73–1.32)	0.06
Student	0.82 (0.67–1.01)	0.63
Own business	1.15 (0.64–2.06)	
**Family members**		
02-Apr	1	0.77
04-Jun	1.03 (0.83–1.29)	0.83
> 6	0.97 (0.77–1.23)	0.61
Single	0.91 (0.62–1.33)	
**Age**		
15–29 year	1	0.71
30–44 year	1.04 (0.85–1.28)	< **0.001**
45–59 year	1.57 (1.22–2.01)	0.35
> 60 year	1.21 (0.81–1.79)	

Data presented odd ratio (95% CI).

*p* value < 0.05, 0.01 consider significant.

Majority of the respondents didn't do group physical activity at home with their families (84.7%), and most didn't try to improve their physical activity habits during the quarantine (not shown in tables).

Table 5 shows the comparison in eating habits before and during the lockdown. Overall significant changes in the patterns of food consumption were observed in participants overtime. The percentage of participants who were always interested in healthy diet significantly decreased during the quarantine than before (37% vs 33%; *p* < 0.001). In parallel, there was a significant decrease in the percentage of participants who always consumed fast food during the quarantine (56% vs 52.9%; *p* = 0.007). The percentage of participants who often consume snacks between meals increased during quarantine (27.4% vs 29.4%, *p* < 0.001). In contrast, the percentage of participants who never consumed fresh fruits and vegetables significantly increased during home quarantine (2.4% vs 3.7%; *p* = 0.019).

**Table 5:** Dietary information in all subjects.

Dietary information	Before					During					*p* value
	Never	Hardly	Sometime	Often	Always	Never	Hardly	Sometime	Often	Always	
Interest in healthy diet	43 (2.2)	203 (10.3)	312 (15.9)	680 (34.6)	727 (37.0)	61 (3.1)*	169 (8.6)*	332 (16.9)	754 (38.4)**	649 (33.0)**	< 0.001
Consumption of…											
Fast food	30 (1.5)	119 (6.1)	263 (13.4)	452 (23.0)	1101 (56.0)	72 (3.7)**	110 (5.6)	265 (13.5)	478 (24.3)	1040 (52.9)**	< 0.001
Coffee from coffee shops	56 (2.8)	128 (6.5)	301 (15.3)	702 (35.7)	778 (39.6)	65 (3.3)	132 (6.7)	318 (16.2)	750 (38.2)*	700 (35.6)**	< 0.001
Snacks between meals	181 (9.2)	603 (30.7)	395 (20.1)	539 (27.4)	247 (12.6)	188 (9.6)	535 (27.2)	432 (22.0)**	578 (29.4)	232 (11.8)	0.007
Homemade food	238 (12.1)	644 (32.8)	423 (21.5)	395 (20.1)	265 (13.5)	329 (16.7)**	612 (31.1)	403 (20.5)	378 (19.2)	243 (12.4)*	< 0.001
Fresh fruits and vegetables	47 (2.4)	231 (11.8)	390 (19.8)	777 (39.5)	520 (26.5)	73 (3.7)**	223 (11.3)	391 (19.9)	784 (39.9)	494 (25.1)	0.019

Data presented n (%).

* and ** represented *p* value significant at < 0.05 and 0.01 level using Cochran’s Q test and McNemar’s test.

## Discussion

The present results of the study, while showing statistically significant changes in the physical and dietary behaviors before and during lockdown, represent a modest deviation in lifestyle in response to the lockdown imposed. Small but statistically significant changes in percentages are commonly observed in survey studies with large sample sizes, and these results should be interpreted with caution. The study nevertheless observed a negative effect of home quarantine on physical activity. Additionally, an unhealthy pattern of food consumption (the type of food, eating more frequently) was exhibited. Despite endorsements that home quarantine should not hinder people from practicing physical activity^25^, our results indicated that the opposite occurred for some respondents. The reported decrease in daily walking was a shift during quarantine, most likely due to the increased confinement time that people were required to follow, effectively increasing sedentary behavior and its associated risks^26^.

The present survey results agree with recent studies showing that lockdowns during a pandemic can adversely influence lifestyle activities worldwide, as well as participation in sports and physical activity^27,28^. Precautions during COVID-19 have decreased physical activity overall and availability of exercises. Despite the increased physical activity guidance and courses presented on social media^29,30^, the current results showed that it was not possible for participants with home activities to preserve their regular physical activity patterns adequately. It was previously demonstrated in China that different socio-economic factors and regional policies were linked to changes in physical activity^31^. Such influences should be considered while planning and endorsing physical activity interventions during COVID-19 pandemic. Recently, it was established that individuals exhibit a larger use (15%) of data and communications technology during the quarantine period^32^. Hence, future physical activity intervention during pandemic can be based on communications technology solutions fitness apps to promote an active and healthy lifestyle during quarantine.

The present survey results also showed that contrary to the World Health Organization (WHO) guidance^33,34^, many people, but not all, responded by changing their eating behaviors, by greater consumption of snacks between meals. Regarding dietary behaviors, there seems to be no single behavioral problem. The negative changes in the majority of eating behaviors could be attributed to eating out of anxiety or boredom^35^, a dip in motivation to participate in physical activity or maintain healthy eating^36^ or an increase in mood-driven eating^30^. Alternative support for motivation during home quarantine may be sourced from assistive technologies such as apps, streaming services, and social media. In order to counteract poor dietary behaviors, meal planning and controlling food composition and meals’ caloric content using information and communication technology-based solutions such as *m*health and nutrition apps may be the best approach to combating unhealthy eating habits while in quarantine^32,35^.

The present study results should be utilized for further research and development in public health campaign in the event of future lockdowns. During the COVID-19 pandemic restrictions, it was proposed that breaking up prolonged sitting by simple means, for instance 30 min periods of shifting between sitting and standing, can significantly increase the energy expenditure, consequently stimulating metabolic health in terms of glycemic control both in diseased and healthy people^37,38^. Individuals who have no history of eating disorders are encouraged to maintain healthy eating habits even during lockdowns by following these dietary behaviors: (a) reducing total number of meals, (b) good quality meals (e.g., more fresh vegetables, good quality protein source, avoiding refined and high glycemic foods), and (c) adopting intermittent or long fasting periods (i.e., more than 12 h)^29,32^. Further research should address (i) insight into subpopulations for the development of interventions to address their needs, (ii) interference of diet and physical activity behaviors to improve interventions, and (iii) identification of conditions for successfully maintaining a healthy lifestyle before as well as during isolation.

The authors acknowledge several limitations. The use of a self-reported questionnaire is subject to recall bias. Majority of the respondents answered the questionnaire during Ramadan, where fasting is mandatory for all able-bodied Muslims. This may have somehow influenced the responses of participants, though the questionnaire was made clear that it was entirely about the lockdown. Lastly and as mentioned previously, small changes in percentages can translate to statistically significant differences in large-scale studies and this should be considered whether such statistical difference have clinical implications.

## Conclusions

The present survey results showed modest and acute adverse consequences of home quarantine as reflected by a more sedentary lifestyle and altered eating habits by some residents of Saudi Arabia. The present data support the development of recommendations for physical activity and nutrition to maintain healthy lifestyle during lockdowns utilizing social media platforms to develop health behavior support as well as identifying populations that are more likely to negatively respond to lockdowns by practicing unhealthy lifestyle behavior.

## Supplementary Information

Supplementary Information.
