# Supplementary material for: Effects of home quarantine during COVID-19 lockdown on physical activity and dietary habits of adults in Saudi Arabia
Source: Sci Rep. 2021 Mar 15;11:5904. doi: 10.1038/s41598-021-85330-2 (PMC7961137; doi:10.1038/s41598-021-85330-2)
Supplement: Supplementary file 1 — Supplementary Information. [file 41598_2021_85330_MOESM1_ESM.pdf]

King Saud University

Lifestyle and health habits during COVID19 Lockdown among Adults in Saudi Arabia

إن الانتشار السريع لفيروس كورونا COVID 19 وماتبعه من إجراءات احترازية والحظر الصحي والتباعد الاجتماعي قد يؤثر على أسلوب حياتنا ونشاطنا البدني لذا فان هذه الدراسة الاستطلاعية تهدف لدراسة التغيرات في أسلوب الحياة والعادات الصحية أثناء فترة الحجر الصحي والحظر قبل رمضان وفي رمضان بسبب فيروس كورونا المستجد.

يمكنك المشاركة في الاستبيان اذا كنت مواطن او مقيم بالغ. كل ماعليك هو قراءة كل عبارة بدقة والإجابة على كل سؤال بوضع علامة أمام الاختيار الذي ينطبق عليك, فضلا لاتترك عبارة دون الرد عليها وتذكر الوقت الذي تقضيه في ملء الاستبيان هو مساهمة منك للمساعدة في الوضع الحالي ف"كلنا مسؤول".

المعلومات سرية ولا تستخدم الا في أغراض البحث العلمي فقط. شكرا على حسن التعاون والإجابة.

The rapid spread of COVID-19 and the strict precautionary measures that followed such as lockdowns, curfews and social distancing, have affected our over-all way of life. This survey aims to study the changes in lifestyle and health habits during the lockdown period before and during Ramadan to contain the spread of COVID-19 in the Saudi community.

You can participate in this survey if you are an adult citizen or resident. Please read each statement carefully and mark the choice that best applies to you. Please do your best to not leave any question/statement blank. The information you provide will remain confidential and will be used only for research purposes.

Your input in this questionnaire is valuable and appreciated.

We are all responsible.

King Saud University

Lifestyle and health habits during COVID19 Lockdown among Adults in Saudi Arabia

Gender الجنس: [ ] Male ذكر [ ] Female انثي

Marital Status الحالة الاجتماعية: [ ] Single اعزب [ ] Married متزوج [ ] Widow/er أرمل [ ] Divorced مطلق

Nationality الجنسية: [ ] Saudi سعودي [ ] Non-Saudi غير سعودي

Age (years) العمر: ____________

Which city do you live? في أي مدينة تسكن؟: ____________

Education التعليم: [ ] High School ثانوي [ ] Bachelor جامعي [ ] Master ماجستير [ ] PhD دكتوراة

Monthly income (SAR) الدخل الشهري:

[ ] Less than 5000 اقل من [ ] 5001-7000 [ ] 7001-16000
[ ] More than 16000 أكثر من

Employment Status الحالة العملية:

[ ] Employed موظف [ ] Unemployed غير موظف [ ] Student طالب [ ] Self-employed أعمال حرة [ ] Farmer مزارع

Work venue during daytime curfew العمل وقت الازمة

[ ] I travel to my workplace أذهب إلى مقر عملي [ ] Home أعمل من المنزل
[ ] I don't work لا أعمل

How many members in your family live with you? كم عدد أفراد أسرتك؟

[ ] None (lives alone) [ ] 2 - 4 [ ] 4 - 6 [ ] More than 6 أكثر من

Were you diagnosed with COVID 19? هل أنت مصاب بمرض الكورونا؟

[ ] Yes نعم [ ] No لا

Any of your family members diagnosed with COVID 19?

هل تم تشخيص أحد أفراد أسرتك بالكورونا؟

[ ] Yes نعم [ ] No لا [ ] I do not know لا أعلم

What COVID 19 symptoms did you or your family member have?

ماهي اعراض الكورونا التي ظهرت لديك او لدى افراد اسرتك ؟

[ ] Fever الحرارة [ ] Cough السعال [ ] Tiredness جفاف الحلق

[ ] Shortness of breath ضيق تنفس [ ]الخمول weakness

[ ] All the above جميع ما ذكر

[ ] أخرى الرجاء ذكرهاOthers (Please mention)

…………………………………………………………………………..

I will isolate myself at the first genuine sign of COVID-19 symptoms.
كنت سأعزل نفسي عند ظهور اول علامة للاصابة بكورونا

[ ] Yes نعم [ ] No لا [ ] I do not know لا أعلم

I use tissues or elbow to cover my mouth when I cough or sneeze since the outbreak began.
منذ تفشي الكورونا وأنا دائما أستخدم المنديل او المرفق عند السعال والعطاس

[ ] Yes نعم [ ] No لا [ ] I do not know لا أعلم

Before lockdown, I was physically active. قبل الحظر امارس الرياضة واهتم بالنشاط البدني

[ ] Yes نعم [ ] No لا [ ] I do not know لا أعلم

Statement<br>العبارة	Highly agree<br>أوافق بشدة	Agree<br>أوافق	Neutral<br>محايد	Disagree<br>لا أوافق	Highly Disagree<br>لا أوافق بشدة
Since the pandemic started… (check what applies) **(ضع علامة أمام الخيار اللذي ينطبق عليك)..منذو بداية الجائحة**					
**My relationship with my family improved**<br>**العلاقات الأسرية داخل الأسرة اكثر ارتباطا بعد أزمة كورونا**					
**Me and my family became more hygienic** (e.g., frequent hand wash, cleaning house, disinfecting purchased items, etc…)					

أنا وأسرتي نتبع الارشادات والاحترازات من العدوى بكورونا(مثل غسل اليدين باستمرار شراء معقمات. تنظيف المنزل)					
**I cared about improving my immunity** أهتم بتقوية مناعتي للحد من الاصابة بالعدوى من كورونا					
**(For smokers) I quit smoking** في حالة انني مدخن أقلعت عن التدخين حتى لا أصاب بالعدوى					
Followed a **healthy diet** اتبع نظام صحي منوع					

Statement العبارة	Yes نعم	No لا	Dose الجرعة	Daily يوميا	Weekly اسبوعيا
I take the following supplements during lockdown to boost my immunity (check what applies) اتناول المكملات الغذائية التالية أثناء الحظر لتقوية مناعتي. اختار/ي الاجابات التي تنطبق عليك					
Multivitaminsمجموعة فيتامينات					
Vitamin D فيتامين د					
Selenium السيلنيوم					
Vitamin C فيتامين ج					
Zinc الزنك					
Others (please mention) أخرى الرجاء ذكرها	……………………..				

During the lockdown period I…... أثناء فترة الحجر والحظر

- Suffered from anxiety **أعاني من القلق**

[ ] Yes نعم [ ] No لا [ ] Sometimes احيانا

- Suffered from depression **أعاني من الاكتئاب**

[ ] Yes نعم [ ] No لا [ ] Sometimes احيانا

- Slept more. **زادت ساعات النوم**

[ ] Yes نعم [ ] No لا [ ] Sometimes احيانا

- Suffered from insomnia. اشعر باضطراب النوم بسبب التوتر من فيروس كورونا

[ ] Yes نعم [ ] No لا [ ] sometimes احيانا

- Slept ____ hours [ ] continuously [ ] intermittently (interrupted)

hour كم يبلغ عدد ساعات النوم تقريبا بعد كورونا متواصله [ ] متقطعة [ ] --------ساعه

- Exposed myself to sunlight to get Vitamin D

أتعرض لأشعة الشمس أثناء جائحة كورونا للحصول على فيتامين د

[ ] Yes نعم [ ] No لا [ ] Sometimes احيانا

How many minutes do you expose yourself to sun light during lockdown?

كم دقيقة تقريبا تتعرض للشمس أثناء جائحة كورونا

[ ] 10-15 min/day 15-10 دقيقه يوميا [ ] 20-30 min/day 30-20 دقيقه يوميا

[ ] 10-15 min/day, three times/week 15-10 دقيقه اسبوعيا

[ ] 20-30 min/day, three times/week 30-20 دقيقة اسبوعيا

[ ] I don't expose myself to sunlight لا اتعرض للشمس

How many glasses of water you drink during lockdown? ___________ glasses

كم عدد أكواب الماء التي تشربها خلال اليوم أثناء جائحة كورونا

كوب ....

الطول Height [-------cm\سم]

Before lockdown قبل الحظر وجائحة كورونا

Weight (kg) الوزن كجم [---- كجم\kg]

During/after Ramadan بعد الحظر وجائحة كورونا

Weight (kg) الوزن كجم [------ كجم\kg]

King Saud University

Lifestyle and health habits during COVID19 Lockdown among Adults in Saudi Arabia

The questions below apply during the lockdown period Not apply on Ramadan

يرجى مراعاة أن هذه الأسئلة لا تشمل نمط الحياة في شهر رمضان المبارك

Statement<br>العبارة	Never<br>اطلاقاً	Hardly ever<br>نادرا	Sometime<br>احيانا	Fairly often<br>كثير من الاحيان	Always<br>دائماً
Interest in healthy diet<br>الاهتمام بالنظام الغذائي الصحي					
Consumption of fast food<br>استهلاك الوجبات السريعة					
Consumption of coffee from coffee shops<br>استهلاك القهوة من المقاهي					
Consumption of snacks between meals<br>استهلاك الوجبات الخفيفة بين الوجبات					
Consumption of homemade food<br>استهلاك الطعام المنزلي					
Consumption of fresh fruits and vegetables<br>استهلاك الفواكه والخضروات الطازجة					
I did not change my diet.<br>لم تتغير عاداتي الغذائية					
I ate more junk food.<br>زاد استهلاكي للحلويات والمسليات					
I followed a strict weight-loss diet<br>أتبع حمية غذائية لانقاص الوزن					
My family and I ate more homemade food.<br>تغير نمط السلوك الغذائي لدي ولدى أسرتي فزاد الأهتمام بالأكل الصحي واعداد الطعام المنزلي					
Food delivery apps increased fast food consumption.<br>وجود التطبيقات الخاصة بالتوصيل زاد من استهلاكي والاسرة للوجبات السريعة أثناء جائحة الكورونا					

Physical Activity during lockdown النشاط الحركي والرياضة أثناء الحظر والحجر

Statement<br>العبارة	Never<br>اطلاقاً	1-2 days/week<br>يوم-يومان/اسبوع	3-4 days/week<br>ثلاثة-اربعة ايام/اسبوع	>4 days/week<br>اكثر من 4 ايام/اسبوع
**Daily Walking**<br>أمارس المشي يوميا أثناء جائحة كورونا				
**Home equipment physical activities with weights**<br>أمارس رياضة منزلية بالأجهزة الرياضية أثناء جائحة كورونا				
**Swimming**<br>أمارس رياضة السباحة أثناء جائحة الكورونا				

	Yes	No
**I do physical activity with my family.**<br>امارس نشاط بدني مع الأسرة واشجعهم على ذلك		
**Me and my family had an increased interest in physical activity**<br>تغير نمط السلوك الصحي لدي ولدى أسرتي فزاد الأهتمام بالنشاط البدني		

King Saud University

Lifestyle and health habits during COVID19 Lockdown among Adults in Saudi Arabia

The questions below apply during lockdown in Ramadan.

يرجى مراعاة أن الأسئلة التالية هي خلال فترة الحظر بسبب فايروس كورونا وفي أيام شهر رمضان المبارك فقط

Statement<br>العبارة	Never<br>اطلاقاً	Hardly ever<br>نادرا	Sometime<br>احيانا	Fairly often<br>كثير من الاحيان	Always<br>دائماً
**Interest in healthy diet**<br>الاهتمام بالنظام الغذائي الصحي					
**Consumption of fast food**<br>استهلاك الوجبات السريعة					
**Consumption of coffee from coffee shops**<br>استهلاك القهوة من المقاهي					
**Consumption of snacks between meals**<br>استهلاك الوجبات الخفيفة بين الوجبات					
**Consumption of homemade food**<br>استهلاك الطعام المنزلي					
**Consumption of fresh fruits and vegetables**<br>استهلاك الفواكه والخضروات الطازجة					
**I did not change my diet.**<br>لم تتغير عاداتي الغذائية					
**I ate more junk food.**<br>زاد استهلاكي للحلويات والمسليات					
**I followed a strict weight-loss diet**<br>أتبع حمية غذائية لانقاص الوزن					
**My family and I ate more homemade food.**<br>تغير نمط السلوك الغذائي لدي ولدى أسرتي فزاد الأهتمام بالأكل الصحي واعداد الطعام المنزلي					
**Food delivery apps increased fast food consumption.**<br>وجود التطبيقات الخاصة بالتوصيل زاد من استهلاكي والاسرة للوجبات السريعة أثناء جائحة الكورونا					

Physical Activity during lockdownالنشاط الحركي والرياضة أثناء الحظر والحجر

Statement<br>العبارة	Never<br>اطلاقاً	1-2 days/week<br>يوم-<br>يومان/اسبوع	3-4 days/week<br>ثلاثة-اربعة<br>ايام/اسبوع	>4<br>days/week<br>اكثر من 4<br>ايام/اسبوع
**Daily Walking**<br>أمارس المشي يوميا أثناء جائحة كورونا				
**Home equipment physical activities with weights**<br>أمارس رياضة منزلية بالأجهزة الرياضية أثناء جائحة كورونا				
**Swimming**<br>أمارس رياضة السباحة أثناء جائحة الكورونا				

	Yes	No
**I do physical activity with my family.**<br>امارس نشاط بدني مع الأسرة واشجعهم على ذلك		
**Me and my family had an increased interest in physical activity**<br>تغير نمط السلوك الصحي لدي ولدى أسرتي فزاد الأهتمام بالنشاط البدني		
